# Personal and Environmental Predictors of Aggression in Adolescence

**DOI:** 10.3390/brainsci11070933

**Published:** 2021-07-15

**Authors:** Claudia A. Robles-Haydar, Marina B. Martínez-González, Yuliana A. Flórez-Niño, Luz M. Ibáñez-Navarro, José J. Amar-Amar

**Affiliations:** 1 Department of Social Science, Universidad de la Costa, Barranquilla 080001, Colombia; crobles@cuc.edu.co (C.A.R.-H.); yflorez1@cuc.edu.co (Y.A.F.-N.); libanez@cuc.edu.co (L.M.I.-N.); 2 Department of Psychology, Universidad del Norte, Barranquilla 080003, Colombia; jamar@uninorte.edu.co

**Keywords:** big five personality traits model, childrearing, disruptive behavior, moral disengagement, mother rejection, structural equation modeling, values

## Abstract

This study aims to find causal factors of aggression in a group of Latino adolescents to achieve a greater understanding of human nature, taking into account personal and contextual variables. The fundamental hypothesis is that moral disengagement, personality traits, self-esteem, values, parenting, sex, and socioeconomic situation can function as possible casual factors of aggression in adolescents. The study examined the variables using the structural equations model (SEM) to determine causal factors of aggression in a sample of 827 adolescents (54% men and 46% women) between 11 and 16 years of age. According to the scientific literature review, sociodemographic, personal, and familiar variables were included in the causal model. The influence of the variables occurred in two ways: one that inhibits aggression and the other that reinforces it. The results are discussed based on identifying protective and risk factors against aggression: biological sex and values of conformity and transcendence as aggression’s inhibitors and, on the other hand, openness, moral disengagement, and leadership values as the most important predictors of aggression.

## 1. Introduction

Aggression is considered a behavior whose objective is to cause harm to another person [1]. The physical forms of aggression are motor behaviors that cause bodily harm, and the verbal forms can be direct and indirect, such as offensive comments, rumors, and nagging [2]. During adolescence, more intense relationships with aggression have been found, and it is in adolescence where criminal trajectories usually begin, and defiant and antisocial behaviors can be generated [3].

Sex represents a sociodemographic variable frequently associated with aggression, and there is some consensus stating that it is higher in men than in women [4,5]. The reasons for these differences are not entirely clear [6]. However, much has been said about evolutionary inheritance, the biological aspects of sexual differences, and care or socialization practices around the dimensions of masculinity and femininity [7]. In addition, some studies agree that children and young people from violent communities show more significant risks of developing criminal or antisocial behaviors than those in an enriched environment [8,9,10].

On the other hand, values are defined as subjective and emotional beliefs, and motivational constructs, representing what is important in people’s lives. They guide the choice and evaluation of behaviors and events, essential in recognizing the motivations that underlie decision-making and reflection on human behavior [11,12]. This variable has been frequently related to moral judgment and prosocial behavior [13]. Values can be classified as those that regulate the expression of personal characteristics (self-direction, hedonism, achievement, power, stimulation) versus values that regulate relationships with others or those that are oriented to transcendence (universalism, benevolence, tradition, conformity, and security) [14]. There is evidence that values such as benevolence, universalism, and security positively affect personal development, while values such as power and achievement could be related to some difficulties such as depression, stress, and aggression [15,16,17].

Personality is understood as the individual and lasting attributes and inclinations that transmit a sense of identity, integrity, and singularity [18]. According to the Big Five theory, personality traits as kindness, tenacity or awareness, and emotional stability have predictive power on aggressive and antisocial behaviors [19]. Therefore, these behaviors would be modulated by a constellation of low scores in the traits of kindness, tenacity, and emotional stability [18,19].

Another critical aspect to understand the causes of aggression could be self-esteem. Low self-esteem predicts significant psychological imbalances, including aggression and violence [20,21]. However, there is also evidence to support the opposite: some research indicates that violent behavior is mainly related to high self-esteem [22,23]. Furthermore, the most violent criminals and the most hostile nations in the world are characterized by their high levels of self-esteem [22]. In this sense, both high and low self-esteem are probably related to aggression [24].

The relation of childrearing with aggression is taken into account. Support and affection refer to the warmth in parent–child interactions. These are observed in acceptance and tenderness, physical proximity, containment, and negative pole due to rejection [25,26]. The understanding of rejection is related to negative feelings such as anxiety, insecurity, low self-esteem, dependence, and destructive emotions such as anger and emotional insensitivity in children and adults [26]. The dimension of control implies authority and different disciplinary strategies to guide children’s behavior [27]. There are findings on the adverse effects of physical punishment and its relationships with anxiety and aggression in children and adolescents [28,29,30]. Likewise, studies on the effects of inductive discipline, where the parental figure guides the child in reflecting on the repercussions that actions have for others, show positive relationships with prosocial behavior and the internalization of the norm [8,31].

Moral disengagement (MD) has been studied as a predictor of aggression and crime [32,33], bullying [34,35,36] and cyberbullying [37], aggression in young people [4,38,39,40], intimate partner violence [41,42], and terrorism, among others [43,44,45]. Moral disengagement is conceptually defined as the psychological process through which self-reactions are disconnected from inhuman behavior [46], allowing inhuman behaviors to be carried out with little or no self-reproach [47]. MD is present in all people as a propensity to evoke restructuring cognitions of harmful behaviors, making the subject perform actions that are harmful to others with little anguish and guilt [48,49].

The present study aims to provide an integrative view of the multi-causal relationships that affect aggression in Latino adolescents where behavior, environment, and personal factors operate interactively to elucidate the causes of human behavior. We expect to contribute to the prevention and mitigation of the negative consequences of aggression and attempt to achieve a greater understanding of human nature and its potentialities. The fundamental hypothesis is that moral disconnection, personality traits, self-esteem, values, rearing, sex, and socioeconomic situation are possible causes of aggression in adolescents. In this sense, it can be hypothesized that the most critical factors for the prediction of aggression are as follows: the personality trait of emotional instability, moral disconnection, self-improvement values (power and achievement), paternal and maternal rejection, discipline based on punishment, belonging to the male sex, and living in a low socioeconomic level. Likewise, those variables that decrease aggressive behaviors are as follows: personality traits of conscience and kindness, self-transcendence values (universalism and benevolence), inductive discipline, and belonging to the female sex. Self-esteem is expected to influence aggression.

## 2. Materials and Methods

The participants were contacted from four schools in Barranquilla (Colombia) with different socioeconomic conditions. The determination of socioeconomic conditions was obtained according to the classification of the National Statistical System (DANE), which classifies residential properties for the allocation of subsidies and differential charges for home public services in Colombia.

The institutional authorities and teachers sent the information to parents, promoted students’ participation in the research, and obtained informed consent documents.

The following inclusion and exclusion criteria were maintained for the sample.

Inclusion criteria:

- Be between the ages of 11 and 16.
- Consent to participation in the study (parents and adolescents).

Exclusion criteria:

- Reside in towns outside the Barranquilla city.
- Cognitive conditions or language difficulties that affect the correct completion of the questionnaire.

The procedure was approved by the Universidad del Norte ethical committee (approval code 146) and conducted following the Helsinki Declaration (revised in Brazil, 2013). The participants completed the questionnaires in their classrooms for 100 and 180 min. During the session, they were accompanied by a psychologist and a teacher. The data were collected anonymously.

### 2.1. Participants

A total of 827 volunteer adolescents participated in this research. Owing to the characteristics of the population and the difficulties in accessing schools, the sampling was non-probabilistic. See Table 1 for sociodemographic information of the participants.

The adolescents were men (54%) and women (46%) with a mean age of 13.6 years (SD = 2) from different socioeconomic levels of the city. A higher proportion were participants from a medium socioeconomic level (47.9%), followed by those from a low level (37.6%), and a lesser proportion from a high socioeconomic level (14.5%).

### 2.2. Measurements

The Moral Disengagement Scale [50] is a 32-item questionnaire to measures children’s proneness to moral disengagement. The scale allows a score per dimension according to the eight mechanisms of MD and a global score of DM. The scale is Likert-type and is rated in a range of five points: (1) not at all agree to (5) totally agree. The 32-item version is one of the most used in cross-cultural research. Likewise, its reliability levels have been greater than 0.82 [51,52,53,54]. In the present sample, a McDonald’s Omega of 0.94 was obtained.

The Big Five Questionnaire short version [55] is a reduced version of the Big Five Questionnaire for Children BFQ-C [56]. It consists of 30 items and contains five subscales for each personality trait. Each subscale includes six items, rated on a five-point Likert scale: from 1 (almost never) to 5 (almost always). The reliability levels oscillate between 0.75 to 0.82, showing a good internal consistency [55]. In the present study, the results for McDonald’s Omega in each of the factors were above 0.7, which is considered good, except in factor 5 (openness), where the values were 0.65, indicating acceptable reliability.

The Rosenberg Self-Esteem Scale is one of the most used instruments evaluating this construct. It measures a single global dimension of self-esteem through 10 items on a Likert scale, whose alternatives range from 1 point (strongly disagree) to 4 points (strongly agree). The scale has been used in more than 53 countries and has adequate psychometric properties, with reliability levels greater than 0.70 [57,58]. In the present sample, a good level of reliability was obtained (Ω = 0.88).

The Portrait Values Questionnaire (PVQ) is a questionnaire developed by Schwartz [11] to measure ten universal values: self-direction, stimulation, hedonism, achievement, power, security, conformity, tradition, benevolence, and universalism in the adolescent population. The questionnaire consists of 40 items presenting a description of the wishes or aspirations of a hypothetical person, those that implicitly affirm a value, for example, “Having new ideas and being creative is important for him/her. He/she likes to do things originally” (Self-direction) [12]. The scale is Likert-type with six response alternatives, where 1 point is equivalent to the highest perception of similarity with the character (he/she looks a lot like me) and 6 points to the greatest perception of disparity (he/she does not look like me at all), so the score is inverted [16]. Regarding the overall internal consistency of the sample, a McDonald’s Omega of 0.96 was obtained.

Child–Parental Acceptance–Rejection Questionnaire (PARQ-C) measures young people’s perceptions about parental upbringing [59]. It has two identical versions, one for each parent, with 29 items grouped into five subscales: warmth/affection, hostility/aggression, indifference/neglect, undifferentiated rejection, and control. It is a Likert-type scale, with response alternatives ranging from 1 (almost never) to 4 (every day). It allows obtaining scores for each subscale and grouping the scores in a single bipolar dimension of perceived acceptance–rejection [60]. The instrument has been used in more than 500 studies, with excellent psychometric properties [39,60]. The Spanish adaptation’s reliability values oscillate between 0.71 and 0.92 for the father version and between 0.72 and 0.85 for the mother version [60]. Therefore, both versions of the PARQ-C have a good and excellent internal consistency (Ω = 0.90 for the mother and Ω = 0.93 for the father) in this study.

Discipline Interview Children and Adolescents’ version measures how frequently parents use different disciplinary strategies in raising their children [61]. It has 18 items and four subscales: positive discipline, physical discipline, verbal discipline, and induction of guilt. The scale is Likert-type with response alternatives ranging from 1 (never) to 5 (almost every day). Their reliability levels range between 0.51 and 0.72 for each of the subscales [28,62]. Regarding the global internal consistency of the sample, the results were Ω = 0.93 for the maternal version and Ω = 0.94 for the paternal version.

Physical and Verbal Aggression Scale AFV measures aggressiveness in children and adolescents through two subscales: physical and verbal aggression, including a global aggression score [63]. The questionnaire consists of 20 items, five of which correspond to control statements not scored in the final grade. Thus, the physical aggression subscale has eight items, and the verbal aggression subscale has seven. The response alternatives are from 1 point (almost never) to 5 points (very often). The Hispanic version presents adequate internal consistency, with test reliability coefficients 0.84 and re-test 0.70 [64]. In addition, an excellent level of reliability was obtained in the present research, with a McDonald’s Omega of 0.93.

Finally, we used a sociodemographic questionnaire developed for this study, including age, gender, family structure, and socioeconomic level.

### 2.3. Statistical Analysis

We carried out an exploratory data analysis using regression, case suppression, and frequency analysis using SPSS 22. Next, an analysis of structural equations was carried out through the AMOS v16 program. From the model, the path coefficients or Beta (β) coefficients were analyzed, for 99% [*] and 95% [**] confidence; significance values lower than 0.05 were considered relevant. Thus, the direction effects were established, either excitatory (positive sign) or inhibitory (negative sign), and their nature, direct on the variables of interest, or that their effect was mediated, through others or another variable of the model [65].

Subsequently, the indirect effects on the endogenous variable of aggression were determined. For this, the Beta (β) pathway coefficients were multiplied, from the first variable, through the intermediate variable, to the final one, obtaining the total effect or total Beta (βtotal). Then, all the inhibitory effects were added. We carried out the same procedure for the excitatory effects, allowing us to identify the direction of the most significant explanatory power of the model. Finally, the quality indicator of the model transformed into the determination coefficient R^2^ was determined, as well as the Mardia coefficient and the adjustment indicators: [χ^2^], [Sig], RMSEA, ECVI, NCP, CFI, NNFI, NFI, PNFI, and [GL], testing a single model for the variables.

## 3. Results

The following variables were included in the proposed model: sex, socioeconomic level, personality traits (energy, openness, agreeableness, conscientiousness, emotional instability), maternal discipline (positive discipline, punishment, negative discipline, verbal discipline) and paternal discipline (positive discipline, punishment, physical discipline, verbal discipline, guilt induction), and maternal affection (affection, aggression/rejection, control, indifference, trust/friendship) and paternal (affection, aggression/rejection, control)—all exogenous variables. At the next level, endogenous variables were introduced: values (openness to life, leadership, conservation/conformity, transcendence, and power), self-esteem, moral disengagement, and finally aggression.

The assumption of multivariate normality was verified, evaluated from the Mardia kurtosis coefficient (Ku). The standard error (δ) is higher than the criterion of 1.96 (δKu = 93.659), which indicates the absence of multivariate normality. It implies using unweighted least squares (ULS) to interpret the structural equation adjustment indicators. Among the adjustment indicators, we took those whose values do not depend on the comparison between models, as showed in Table 2.

The adjustment indicators were interpreted following the scale established by Hair [66] as follows:

RMSEA (root mean square error of approximation) (between 0.05 and 0.08): it is at a suitable value of 0.05.

Significance of RMSEA or *p*-value of RMSEA (>0.05): the model value was 0.58. So, it was not fulfilled.

NNFI (non-normed fit index) (≈1): these values should tend to 1, so the value of 0.73 that the model produced is considered an acceptable measure.

NFI (normed fit index) (≈1): a value of 0.76 is considered an acceptable measure by the model.

CFI (comparative fit index) (≈1): the value of 0.76 is considered acceptable.

Although there is an adequate adjustment in the model’s variables, it does not explain the total variability of the endogenous variable: aggression.

That is why the results indicate an adequate adjustment in the model’s variables with a good, but not excellent value of the multivariate correlation coefficient (R = 0.43). The significant (β) links were analyzed to explain the causal effects of the independent variables. This is to understand the links and later reflect on which variables should be incorporated in future studies, as well as to contribute to increasing the explanatory capacity of the model.

**Figure 1:**
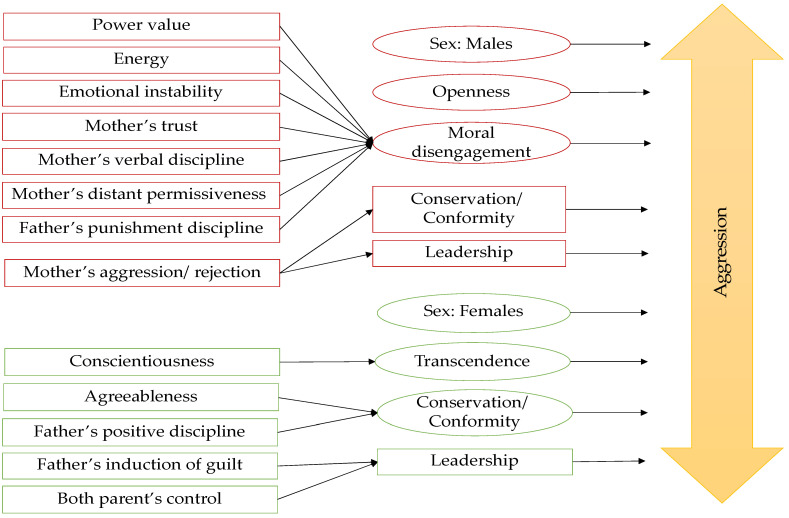
Model to explain aggression in Latin adolescents. The shape used within the path graph represents the variable’s nature if it directly influences aggression (oval shape) or an indirect influence (rectangle). The red color represents the stimulating route of aggression, and the green represents the inhibitory.

Some of the variables evidenced an inhibitory and direct effect on aggression (Figure 1). For the biological sex (β = −0.15), women were less likely to manifest aggressive behaviors than men. The transcendence values (β = 0.11) and conservation/conformity (β = 0.08) were higher in adolescents with lower levels of aggression.

Other inhibitory variables that indirectly affected aggression were the personality traits of conscientiousness (βtotal = −0.03) and agreeableness (βtotal = −0.01). The path from conscientiousness stimulates the value of transcendence (β = −0.25), decreasing aggression (β = 0.11). The route from agreeableness to aggression passes through the value of conservation/conformity (β = −0.19), and from there, it reduces aggression (β = 0.08). Thus, at higher levels of conscientiousness and agreeableness, aggression is lowered.

Continuing with the inhibitory route, the induction of guilt (βtotal = −0.01) as paternal childrearing affects the leadership value, and from there, it inhibits aggression (β = −0.07). Positive discipline used by the father (βtotal = −0.01) affects the conservation/conformity value (β = −0.10), and from there, it affects aggression (β = 0.08). Therefore, the greater the fathers’ use of guilt and induction, the lower their children’s aggressiveness.

Paternal affection was also highlighted in its control dimension (β = −0.01), affecting leadership value (β = 0.09), and from there, decreasing aggression (β = −0.07). Likewise, maternal control (β = −0.0001) passing through leadership value (β = −0.08) affects aggression (β = −0.07), demonstrating the importance of monitoring and limits imposed by the parents on mitigating the aggression.

On the other hand, some variables evidenced a direct and stimulating influence on aggression. The personality trait of openness (β = 0.12) was higher in adolescents with higher levels of aggression. The moral disengagement (β = 0.09) and the leadership value (β = −0.07) showed that those adolescents who seek to maintain a dominant position within the group tend to be more aggressive.

Other variables showed the same stimulating effect on aggression but indirect. The mother’s affection expressed as aggression/rejection (βtotal = 0.02) passes through the leadership value (β = −0.11) and from there to aggression (β = −0.07). In the same way, mother’s aggression/rejection passes through the conservation value (β = 0.13), increasing aggression (β = 0.08). In this way, the greater the maternal aggression/rejection, the greater the identification of young people with the leadership value and less the identification with the value of conservation/conformity, establishing that, at greater maternal rejection, higher levels of aggression are present in adolescents.

The rest of the variables exciting aggression indirectly are those whose route of influence goes through moral disengagement. The distant maternal permissiveness (βtotal = 0.01) and maternal trust (βtotal = 0.01) increase the levels of aggression. Maternal verbal discipline (βtotal = 0.01) and punishment-based discipline from the father (βtotal = 0.0001) are associated with aggressive behaviors in young people. The value of power (βtotal = −0.01) shows that, the greater the pursuit of material success and popularity in adolescents, the higher the levels of aggression. Likewise, the traits of emotional instability (βtotal = 0.01) and energy (βtotal = 0.01) show that, the more emotionally unstable and energetic young people are, the more aggressive they will tend to be.

Finally, variables as socioeconomic level and self-esteem had no significant effect on aggression.

## 4. Discussion

The present work aimed to know the influence of personal and environmental factors on aggression in adolescents. According to the model obtained, we found two ways of influencing aggression: one that inhibits it and another that reinforces it.

As protective factors, the most significant effects were found for sex, the values of transcendence and conformity, as well as the personal trait of conscientiousness. The following factors were found to have lesser effects: the personal trait of agreeableness and aspects of parenting as maternal and paternal control, induction of guilt, and positive discipline applied by the father.

Regarding risk factors, those variables with the most relevant effect were the openness personality trait, moral disengagement, and leadership value. The rest of the variables that reinforce aggression have moral disengagement as a mediator. Then, this variable directly affects aggression and moderates other routes of influence as emotional instability, energy, and power value, as well as maternal breeding factors such as distant permissiveness, trust/friendship, verbal discipline, and rejection.

The literature showed that women present less orientation to aggression than men in all cultures, regarding the inhibitory pathway. These differences in aggressive behavior have been attributed to higher levels of testosterone in males [67]. However, other studies have shown that men and women behave more aggressively when they believe they have higher testosterone levels, regardless of whether they have them or not [68]. Social constructions around masculinity highlight strength, virility, and arrogance in men, stimulating relations of power and submission so that the prevailing masculinity model and its heteronormative framework could constitute an adequate explanation for this phenomenon [4,31].

Personal values such as transcendence and conformity are inhibiting factors of aggression. The first motivates altruism by concern for others and the search for social justice [11,16,69], and the second inhibits actions that can cause harm to others, showing more obedience in individuals who show high levels of conformity [70,71]. It is necessary to mention that conformity also represents the effort to accomplish the norm and moderate actions to maintain the group’s functioning [12]. The interest for not violating social norms or expectations is a kind of subordination to others’ impositions that could be related to avoiding aggressive actions at least in conventional contexts in which the rules are created to protect life and humankind.

Similarly, traits of conscientiousness indirectly reduce aggression. For example, adolescents with high levels of consciousness are trustworthy and self-regulating [63,72]. Likewise, agreeableness indirectly decreases aggression as it stimulates prosocial behaviors by maintaining a positive and empathetic vision of human nature [73].

The effects of parenting are minor around the inhibition of aggression. However, control strategies with norms established and behavior monitoring constitute essential aspects in preventing disruptive behaviors [8]. Positive discipline refers to the use of induction and positive reinforcement to control children’s behavior. Our findings exhibit that occurs when the father uses inductive discipline, calls to reason, and reflection on the consequences of the actions, which have been linked to prosocial behavior and empathy in children [8,9]. On the other hand, guilt induction is the disciplinary technique that promotes shame in children for their bad behavior [28] and has been related to psychological control and conditional love [74,75]. However, the role of guilt in maintaining interpersonal relationships is undeniable as it increases anxiety when harming others and promotes the search for reparation [76]. Guilt is an aspect promoted in all cultures because of its influence on internalizing the norm and regulating behavior [28]. This paradoxical result on the effects of guilt induction as a disciplinary technique may depend significantly on how the parent implements it. In this sense, one of the most important aspects is where the focus of criticism is placed, that is, if the parents decide to criticize the child’s behavior instead of his personality, or if they highlight the negative consequences of the wrongdoing for the victim or the negative feelings that they are experiencing [77].

This disciplinary technique can be effective in preventing aggression, at least in a positive discipline context. However, the fact that it stands out in the paternal bond may be because of the traditional roles in the parenting of Latin American families, where the father is the one who mainly imposes the norm and discipline in the home.

These results are in line with previous research, where aggressive behavior is explained by the combination of attributes that allows the person to exercise adequate control over their impulses, which endow them with empathy and concern for the welfare of others [46,73]. It is not enough to possess the capacities to self-regulate, but rather one must have the motivation and desire to put them into practice [46].

Regarding the combination of variables that stimulate aggressive behavior, the openness trait was the one that had the highest incidence and a direct influence on aggression. Young people with a moderate-disruptive behavior had scores above the average in the openness trait [78]. People characterized by this trait are open to novelty, questioning authority, and social conventions [79], which could lead them to enroll in risky behaviors, especially if they are outspoken, energetic, and emotionally unstable.

Another significant predictor of aggression was moral disengagement, coinciding with previous studies [4,9,38,80,81]. Most acts of human cruelty are the product of a deliberative conscience, in which the person engages in aggressive or malicious behaviors, justifying their actions to reduce feelings of guilt and self-concept [32,49,82].

Finally, and as the last variable whose influence directly affects aggression, is the value of leadership, defined as the importance that people give to having a dominant position within the group, making decisions, and telling others what to do. In this study, maternal aggression increased this value, while the induction of guilt by the father decreased it. In this case, leadership refers to a restrictive aspect of the freedoms of others and the search for control [83]. Autocratic leadership has related to hostile behavior [84].

Among the variables that indirectly incited aggression were significant aspects such as permissiveness, trust, and mother rejection. In correspondence with other variables of the family relationship and the adolescent’s personality characteristics, permissive parenting would produce negative results in development and social performance [85].

A second aspect of the affective relationship with the mother is the trust/friendship dimension. This result reveals a more horizontal relationship, which could reduce the mother’s chances of establishing herself as an authority capable of redirecting adolescent maladaptive behaviors [86]. It is not an inherently negative relationship, although it can become permissive, lacking affection or hostile context as maternal rejection was also evident. Maternal rejection implies feelings of anger, detachment, or resentment towards the child, involving behaviors that intentionally harm them, such as sarcasm, threats, hurtful gestures, slapping, or pinching. In general, rejection has been one of the most studied and documented aspects in research on the influence of parenting on child and adolescent adjustment, with one of its main consequences being feelings of hostility and problems in handling anger and aggression [59].

Most of the studies that have dealt with parenting have been configured around the maternal figure [85]. Few studies have sought to establish differences between the influence of the father and the mother in parenting [87]. Some of these have revealed that maternal rejection would have worse consequences on children’s psychological adjustment and would be involved with greater internalizing and externalizing behavioral manifestations in children and young people [27]. The mother is usually configured as the principal caregiver and giver of affection and tenderness [88]. In this sense, aggression from the mother could have a more harmful effect because she is the figure in charge of giving affection and because of cultural expectations that the children would have about the mother [88]. In the case of discipline, the influence of both parental figures appears, including verbal discipline from the mother and punishment from the father—not physical, but symbolic. Verbal discipline is based on shouting, threats, and insults to control children’s behavior, which is why it has been connected to aggressive behavior in children and young people [89,90]. Likewise, the mother’s verbal discipline was related to the prediction of aggression, and the physical dimensions that did not appear may be because of some limitations in the use of instruments, which make it difficult to establish the limit between discipline and physical abuse [91].

Another critical aspect of the indirect influence on aggression is the value of power relative to ambition and materialism. Power refers to searching for a dominant position within the group and is integrated into the self-improvement dimension, in which personal interests are pursued instead of group interests [12]. In this work, the power dimension includes aspects related to the search for personal success and the desire to obtain lots of money, be admired, and impress others. It is known that the persecution of this type of value motivates selfish behaviors [16,69].

Some authors have found how money and its pursuit have been associated with criminal acts [92], behavioral problems, low social productivity, and in general psychological maladjustment, warning about the devastating consequences that a materialistic view of the world can generate [93,94]. Likewise, people who aspire excessively to the goals of fame and fortune tend to be more narcissistic and Machiavellian [95].

The value of power must be analyzed concerning the amalgam of factors where adolescents’ personality is integrated. Thus, traits as the emotional instability make one prone to the experimentation of negative emotions such as sadness, fear, and anger, with little tolerance to stress and difficulties for self-regulation [96]. In this sense, people with difficulties controlling their negative emotionality would be more likely to act on it [38,97]. In the case of energy, this trait is characterized by the search for social stimulation and dominance and has been linked to high extraversion and high neuroticism with antisocial results [98]. Extraverted people would experience greater difficulties in inhibiting their aggressive impulses; at the same time, their permanent need for stimulation would put them in search of novel and intense situations [98].

Neither self-esteem nor socioeconomic status had any significant impact on aggression. Aggression is not a condition that depends on how much the person likes himself. Baumeister [22] considers that self-esteem relevance has been exaggerated as this construct is not the cause, but rather the consequence of many conditions with which it has traditionally been related. That would explain the lack of congruence between multiple studies in which both low and high self-esteem predicts aggression [20,22,99]. Other studies also affirm that there is no relationship between self-esteem and aggression [100].

Finally, regarding the socioeconomic level, there is no significant influence on aggression. In this case, the most important predictors of aggression have to do with the personal characteristics of adolescents and their interaction with parents, regardless of the socioeconomic level where this interaction occurs.

## 5. Conclusions

This study explored the precursors of aggression in a sample of Latino adolescents, considering environmental and personal variables resulting from the theoretical review. The results allowed us to point out that the most important predictors of aggression were openness, DM, and leadership value. Other significant predictors, with lesser effects, were the relationship with the mother. This parental figure plays an essential role in predicting aggression and becomes a risk factor when described as hostile, permissive, and with little involvement in the child’s life. Concerning the father’s practices, we found that punishments also increased aggression with a lesser effect.

The most significant factors inhibiting aggression were the biological sex, corroborating what has already been exposed by numerous studies, where women have lower levels of aggression than men. Moreover, values of conformity and transcendence were also found as aggression inhibitors. Other factors with lesser effects were personality traits like kindness and conscience, positive or inductive discipline from the father, and the induction of guilt and monitoring from both parents.

In this study, the personal adolescent’s factors such as biological sex, personality, values, and moral disengagement had a more significant impact on aggression than family variables. This phenomenon may be related to a large part of the influence of family passes first towards personal factors and then towards aggression. In this sense, the development of values, for example, depends greatly on socialization in the family environment during early childhood. Later, the values will be introjected and appropriate by the subject.

On the other hand, and contrary to expectations, neither self-esteem nor socioeconomic level significantly influence aggression.

Finally, and through the proposed model, it was possible to explain aggression as a combination of attributes that allow adequate control over impulses and endow empathy and concern for the well-being of others.

### Suggestions for Future Research

In order to further the field of research, it is important to take into account that these findings represent just a modest advance in research on aggressive behaviors. Much remains to be further studied, such as: What are the parents’ differentiated influences in developing aggressive behaviors in adolescents? What other personal or contextual variables can moderate or mediate their development? How culture or other scenarios can affect its development?

The model presented in this study did not explain the total variance of aggression. Therefore, some recommendations could improve the predictive capacity of future alternative models.

First, it is recommended to continue working with variables associated with self-regulation and self-containment, as established in the present study. Therefore, future research should delve into these types of variables and how they can be potentiated. Including variables such as emotional intelligence, the capacity for introspection and experimentation with guilt, and even mirror neuron activity could be clarifying.

Additionally, it is proposed to incorporate dark personality traits, such as narcissism and Machiavellianism, or those that account for cynicism and the ability to manipulate and even question authority.

Research in parenting should continue delving into affection and discipline and the influence of each parent separately. In this regard, it is recommended to work with younger populations and longitudinal studies that reveal how the influence of socializing entities on this variable is developing. Another important aspect is to evaluate the influence of other contexts as the work was only done from within the family, and it could be interesting to explore the influence of the cultural and scholarly context. Finally, sociodemographic factors should continue to be included, albeit as a control measure.

## 6. Limitations

Some of the study’s limitations were non-probabilistic sampling owing to difficulties in entering the schools and subsequent access to the population and the use of questionnaires or self-report measures that could affect the data owing to the social desirability of the participants.

## Figures and Tables

**Table 1:** Sociodemographic characteristics of the participants.

Variable	Description	Percent
Biological sex	Men	54%
	Woman	46%
Socioeconomic level	High	14.50%
	Medium	48%
	Low	37.60%

**Table 2:** Structural equation model fit indicators.

Fit Indicators	[χ^2^]	χ^2^	*p*-Value χ^2^	RMSEA	*p*-Value RMSEA	NCP	ECVI	NNFI	NFI	CFI	PNFI
Aggression prediction model	3381.24	3.04	0	0.05	0.586	2270.24	5.687	0.728	0.76	0.76	0.511

## Data Availability

The data that support our results can be found through directly asking the first author.
